# Methylation of FOXP3 TSDR Underlies the Impaired Suppressive Function of Tregs from Long-term Belatacept-Treated Kidney Transplant Patients

**DOI:** 10.3389/fimmu.2017.00219

**Published:** 2017-03-03

**Authors:** Evelyn Katy Alvarez Salazar, Arimelek Cortés-Hernández, Germán Rodrigo Alemán-Muench, Josefina Alberú, Jesús R. Rodríguez-Aguilera, Félix Recillas-Targa, Victoria Chagoya de Sánchez, Eric Cuevas, Eduardo Mancilla-Urrea, María Pérez García, Guillermo Mondragón-Ramírez, Mario Vilatobá, Ian Bostock, Erick Hernández-Méndez, David De Rungs, Eduardo A. García-Zepeda, Gloria Soldevila

**Affiliations:** ^1^Departmento de Inmunología, Instituto de Investigaciones Biomédicas, Universidad Nacional Autónoma de México, Ciudad de México, Mexico; ^2^Departmento de Trasplantes, Instituto Nacional de Ciencias Médicas y Nutrición Salvador Zubirán, Ciudad de México, Mexico; ^3^Departamento de Genética Molecular, Instituto de Fisiología Celular, Universidad Nacional Autónoma de México, Ciudad de México, Mexico; ^4^Departamento de Nefrología, Instituto Nacional de Cardiología Ignacio Chávez, Ciudad de México, Mexico; ^5^Instituto Mexicano de Trasplantes, Morelos, Mexico

**Keywords:** transplantation, tolerance, belatacept, Cyclosporine A, Treg, suppression

## Abstract

Regulatory T cells (Tregs) are considered key players in the prevention of allograft rejection in transplanted patients. Belatacept (BLT) is an effective alternative to calcineurin inhibitors that appears to preserve graft survival and function; however, the impact of this drug in the homeostasis of Tregs in transplanted patients remains controversial. Here, we analyzed the phenotype, function, and the epigenetic status of the Treg-specific demethylated region (TSDR) in FOXP3 of circulating Tregs from long-term kidney transplant patients under BLT or Cyclosporine A treatment. We found a significant reduction in the proportion of CD4^+^CD25^hi^CD127^lo/−^FOXP3^+^ T cells in all patients compared to healthy individual (controls). Interestingly, only BLT-treated patients displayed an enrichment of the CD45RA^+^ “naïve” Tregs, while the expression of Helios, a marker used to identify stable FOXP3^+^ thymic Tregs remained unaffected. Functional analysis demonstrated that Tregs from transplanted patients displayed a significant reduction in their suppressive capacity compared to Tregs from controls, which is associated with decreased levels of FOXP3 and CD25. Analysis of the methylation status of the FOXP3 gene showed that BLT treatment results in methylation of CpG islands within the TSDR, which could be associated with the impaired Treg suppression function. Our data indicate that analysis of circulating Tregs cannot be used as a marker for assessing tolerance toward the allograft in long-term kidney transplant patients. Trial registration number IM103008.

## Introduction

Kidney transplantation is considered the treatment of choice for patients with end-stage renal failure. The use of calcineurin inhibitors (CNI) such as Cyclosporine A (CsA) and tacrolimus reduce allograft rejection (1); however, the toxic effect produced by the prolonged use of these immunosuppressive drugs, has aimed researchers to develop new drugs that prevent allograft rejection without the side effects of CNI (2). Among them, belatacept (BLT), a human fusion protein combining a modified extracellular portion of cytotoxic T-lymphocyte-associated antigen 4 (CTLA-4) with the constant-region fragment (Fc) of human IgG1, was approved in 2011 by US Food and Drug Administration as therapeutic agent for the prevention of allograft rejection. BLT mediates its function by interacting with costimulatory ligands CD80/CD86 on the antigen-presenting cell (APC), thereby blocking the costimulatory signal (second signal) required for naive T cell activation and resulting in anergy or apoptosis (3). A lower incidence of chronic allograft nephropathy, favoring BLT compared to patients under CsA treatment was documented by 12 months post-transplantation (4, 5). Although, it has been reported an increase of acute rejection episodes and post-transplant lymphoproliferative disorders in BLT-treated patients (4), clinical studies have shown that 7 years after transplantation kidney transplant patients under BLT treatment have a significantly better patient and graft survival, as well as mean estimated glomerular filtration rate than patients under CsA (6).

Regulatory T cells (Tregs) are cells with immunosuppressive capacity that are important in tolerance maintenance after organ transplantation (7). These cells are characterized by the expression of the transcription factor FOXP3, considered the master regulator of their development and function, as well as high levels of surface CD25, and low levels of CD127 (CD4^+^CD25^hi^CD127^lo/−^FOXP3^+^). Therefore, several studies have addressed the potential effects of immunosuppressive therapy on Tregs from transplanted patients.

Some studies have claimed that BLT, rather than acting as immunosuppressor, plays a role as immunomodulator. This was supported by studies showing an increase of CD16^+^IDO^+^ cells and Tregs in peripheral blood and in intragraft FOXP3^+^ cells, proposed to generate a tolerant profile to renal allograft in those patients (8, 9). Other studies showed no differences in the percentage of CD4^+^CD25^hi^ and in FOXP3 levels in the CD4^+^ subpopulation between BLT and CsA patients after 6 months of treatment (10) or showed no long-term negative impact of BLT on circulating Tregs (11). By contrast, other studies reported a lower percentage of CD4^+^CD25^hi^ cells in peripheral blood and a reduced intragraft level of FOXP3 mRNA compared to CsA (12). However, it was argued that the negative effect of BLT might be the result of short-term effect of the basiliximab used in the induction therapy. Therefore, the effect of this drug on Tregs remains controversial [reviewed in Ref. (13)].

As BLT is a costimulation blocker, it could interfere with signaling pathways (CD28 and CTLA-4) important for the development, homeostasis, and function of Tregs (14, 15). As these cells are important in tolerance maintenance after organ transplantation (7), it is necessary to study not only their frequency but also their suppressor function and FOXP3 epigenetic status, in transplanted patients under long-term therapy. Thus, the aim of the present study was to analyze the impact of long-term therapy with BLT or CsA on the phenotype, suppressive function, and the epigenetic status of the FOXP3 TSDR from peripheral Tregs of kidney transplant patients with stable graft function. Our data indicate that analysis of circulating Tregs cannot be used as a marker for assessing tolerance toward the allograft in long-term kidney transplant patients.

## Materials and Methods

### Kidney Transplant Patients

Thirty-five primary kidney transplant patients included in this sub-study participated in the clinical trial BENEFIT (IM103008 study), which continue on their original study drug arm, BLT (*n* = 24) or CsA (*n* = 11). All patients received induction therapy with basiliximab, and adjunctive maintenance therapy with mycophenolate mofetil and prednisone. BLT was administered at a dose of 5 mg/kg every 4 weeks and CsA in an adequate dosage to maintain a blood level of 100–200 ng/dl (current mean blood level of 132.8 ± 40.1, 65.6–189.4). The current daily dose for mycophenolate mofetil and prednisone in both groups was 1 g/day and 5 mg/day, respectively. The daily exposure to mycophenolate mofetil and prednisone was comparable in both groups.

This sub-study was conducted with authorization of Bristol-Myers Squibb (protocol IM103-351) and approval from the Committees Medical Ethics at the Instituto de Investigaciones Biomédicas (UNAM), the Instituto Nacional de Ciencias Médicas y Nutrición Salvador Zubirán (Reference number 1535), the Instituto Nacional de Cardiología Ignacio Chávez, and the Instituto Mexicano de Trasplantes, and performed in accordance with the revised Declaration of Helsinki content, the Declaration of Istanbul, and Good Clinical Practice Guidelines. All patients provided written informed consent to participate in the study.

The transplanted individuals had stable graft function and with no clinical/biochemical evidence of rejection. Transplanted individuals in this sub-study had a mean of 7 years post-transplant. Buffy coat preparations of normal blood donors (control group) were provided by the Blood Bank, Instituto Nacional de Enfermedades Respiratorias, México DF.

### Reagents

Anti-CD127-PECy7, anti-CD25-PECy5, anti-CD45RA-APCH7, anti-CD19-FITC, and anti-CD3-APCCy7 were purchased from BD Biosciences (San Jose, CA, USA). Anti-CD4-PE and anti-CD8-PECy7 were purchased from Tonbo Biosciences (San Diego, CA, USA). Anti-FOXP3-AlexaFluor647 was purchased from Beckman Coulter (Brea, CA, USA). Carboxy fluorescein succinimidyl ester (CFSE) was purchased from Life Technologies (Eugene, OR, USA).

For intracellular staining, FOXP3/Transcription Factor Staining Buffer Set (eBiosciences, San Diego, CA, USA) was used. All staining were performed in RPMI 1640 (Gibco, Carlsbad, CA, USA) with 10% fetal bovine serum (FBS), 2 mM glutamine, 10 mM HEPES, and 10 mM antibiotic-anti-mycotic (Gibco).

### Cell Isolation, Antibody Staining, and Cell Sorting

Peripheral blood mononuclear cells (PBMCs) were isolated from blood of patients or from the buffy coat preparations of healthy donors by standard Ficoll-Paque™ Plus density-gradient centrifugation (GE Healthcare). PBMCs were labeled with anti-CD4, anti-CD127, anti-CD25, and anti-CD45RA monoclonal antibodies, incubated at 4°C in the dark for 20 min. For intracellular staining, cells were permeabilized with 150 μL of fixation/permeabilization solution at 4°C for 12 h. After two washes with permeabilization buffer 1×, PBMCs were incubated with anti-FOXP3 and anti-Helios for 30 min at 4°C in the dark. Samples were acquired with an Attune^®^ Acoustic Focusing Cytometer (Life Technologies) and FlowJo 7.6.2 software (Tree Star, Ashland, OR, USA) was used for data analysis. A total of 100,000 events were acquired for analysis, after gating of lymphocytes based on the FSC/SSC dot plot.

For sorting, 50 × 10^6^ PBMCs were washed once and resuspended in media (RPMI/10% FBS) at 100 × 10^6^ per ml. Cells were incubated with anti-CD4 and anti-CD25 at 4°C for 30 min, washed twice and resuspended in PBS 1×. Labeled cells were sorted using a FACs Aria I sorter (BD Biosciences). For Treg isolation, a CD4^+^CD25^veryhi^ gate was used and sorted cells were collected in media (RPMI/20% FBS), washed once and resuspended in culture media until ready to be plated in the suppression assay.

### T Cell Suppression Assays

As a significant percentage of the patient’s Tregs did not express low levels of CD127, we were unable to use the conventional CD4^+^CD25^hi^CD127^lo^ region to purify Tregs, and instead a CD4^+^CD25^veryhi^ gate (containing >85% of Foxp3^+^ T cells) was used to isolate Tregs from both healthy subjects or kidney transplant patients (see Figure S3 in Supplementary Material). Sorted Tregs were cocultured with CD3^+^ cells (Tresp) from the same individuals. CD3^+^ T cells were isolated by negative selection using a Pan T cell Isolation Kit (Miltenyi Biotec, San Diego, CA, USA) according to the manufacturer’s instructions, then labeled with 2.5 μM of CFSE at 37°C for 15 min, and finally washed three times with RPMI 10% FBS. In all assays, 5 × 10^4^ CFSE-labeled Tresp were cocultured with Tregs at ratios Tregs:Tresp (0:1, 1:2, 1:4, 1:8, and 1:16) in RPMI media supplemented with 10% human AB serum and in the presence of Human Treg Suppression Inspector beads (Miltenyi Biotec) at a bead:Tresp ratio of 1:1. After 5 days, cells were stained with antibodies to CD4 and CD8, and cells were acquired with an Attune^®^ Acoustic Focusing Cytometer and FlowJo 7.6.2 software was used for data analysis. The percentage of proliferating Tresp cells was determined by CFSE dilution and unlabeled CFSE-negative Tregs were excluded. Suppression was calculated as relative inhibition using the following formula: [(Tresp proliferation without Tregs − Tresp proliferation with Tregs)/Tresp proliferation without Tregs] × 100, for each individual.

### Cytokine Production Assay

The levels of the cytokines IL-2 and IFN-γ in the culture supernatants from suppression assay were measured using LEGENDplex bead-based immunoassay KIT (Biolegend), according to the recommended procedure. Briefly, the samples were incubated with a panel of capture beads, then mixed with biotinylated detection antibody and subsequently with streptavidin–phycoerythrin, providing flourescent signals that were quantified on a flow cytometer. The concentrations of the cytokines were determined using a standard curve generated in the same assay. The experiments were performed in quadruplicate and repeated two times.

### DNA Methylation Analysis of the FOXP3 TSDR

Methylation of Treg-specific demethylated region (TSDR) of FOXP3 gene was evaluated in isolated CD4^+^CD25^hi^ from transplanted patients and controls previously used for suppression assays (CD4^+^CD25^veryhi^ gate). As negative controls, CD4^+^CD25^−^CD45RA^+^ T cells from controls were included. Sodium bisulfite modification of genomic DNA was carried out using the EZ DNA Methylation direct Kit (Zymo Research Corp., USA) according to the manufacturer’s protocol. Bisulfite-treated DNA was PCR amplified using the following primers: *p*-5′-TGTTTGGGGGTAGAGGATTT-3′ and *o*-5′-TATCACCCCACCTAAACCAA-3′. Amplified DNA product was gel purified using QIAEX II gel extraction kit (Qiagen, Germany) and cloned into pGEM-T easy vector (Promega). *Escherichia coli* competent cells were transformed with recombinant vector 10 individual positive bacterial colonies were selected from which recombinant plasmid DNA was purified and sequenced with 3500 Genetic Analyzer (Applied Biosystems, USA). Sequences were interpreted using the Bioedit Software 7.2.5 (Ibis Biosciences, USA).

### Statistical Analysis

The statistical analysis was performed using Prism 5.0 software (GraphPad Software, San Diego, CA, USA). Values were expressed as mean ± SEM. The Kolmogorov–Smirnov test was used to evaluate the distribution of each parameter. Only the control group followed a normal distribution. Differences between three groups were calculated using the Kruskal–Wallis test and comparison between two groups were made using the two tailed, Mann–Whitney non-parametric *U* test. A value of *p* < 0.05 was considered significant.

## Results

### Kidney Transplant Patients

Twenty-four patients were included in the BLT group and 11 patients were in the CsA group. Clinic and demographic characteristics of the patients included in the study are shown in Table 1. As observed in this Table, no differences were found in donor and patients characteristics, although patients under BLT treatment maintained significantly better and stable graft function (both serum creatinine and cGFR) compared to patients under CsA. All patients were included at comparable years after transplantation. There were no differences in the total number of HLA mismatches. The control group consisted of a group of nine healthy individuals (eight males and one female) closely matched with kidney transplant patients for age, with a mean of 35 years (24–54 years). Furthermore, the current number of peripheral blood lymphocytes was comparable between both transplant groups (3.2 ± 1.7 × 10^3^ cells/μl blood and 2.5 ± 1.0 × 10^3^ cells/μl blood for BLT and CsA, respectively) and to controls (2.5 ± 0.5 × 10^3^ cells/μl blood).

**Table 1:** **Clinic and demographic characteristics of kidney transplant patients and controls**.

	Belatacept (*n* = 24)	CsA (*n* = 11)	Control (*n* = 9)	*p* value
Age (years)	31.08 ± 10.1 (18–57)	28.9 ± 10.7 (18–50)	35 ± 10.5 (24–54)	0.565
Gender (F/M)	10/14	3/8	1/8	0.478
Donor age (years)	36.12 ± 9.47 (21–54)	37.81 ± 9.34 (23–52)	NA	0.625
Donor gender (F/M)	17/7	6/5	NA	0.451
Haplotypes (2-H/1-H/0-H)	2/14/8	2/6/3	NA	0.755
ESRD cause	Unk: 18	Unk: 9	NA	0.672
	DM: 3	DM: 0		
	GMN: 3	GMN: 2		
1-year SCr post-KT (mg/dl)	1.05 ± 0.19 (0.7–1.4)	1.34 ± 0.27 (0.95–1.83)	NA	0.0011
Current SCr (mg/dl)	1.03 ± 0.304 (0.66–2.03)	1.48 ± 0.43 (0.9–2.1)	NA	0.0012
1-year post-KT cGFR (ml/min) by MDRD	79.01 ± 15.6 (56.1–126.2)	63.81 ± 9.1 (50.9–78.9)	NA	0.0056
Current cGFR (ml/min) by MDRD	79.6 ± 16.5 (40.1–107.7)	56.8 ± 13.6 (38.1–76.1)	NA	0.0004
1-year post-KT serum CsA blood level (ng/ml)	NA	155.9 ± 36.2 (112–228.7)	NA	
Current serum CsA blood level (ng/ml)	NA	132.8 ± 40.1 (65.6–189.4)	NA	
Time since transplantation (years)	7.28 ± 0.91 (5.9–8.67)	7.11 ± 0.83 (5.9–8.22)	NA	0.606
Peripheral blood lymphocytes (cells/μl blood)	3.2 ± 1.7 × 10^3^	2.5 ± 1.0 × 10^3^	2.5 ± 0.5 × 10^3^	0.478

*ESRD, end-stage renal disease; Unk, unknown; GMN, glomerulonephritis; DM, diabetes mellitus; SCr, serum creatinine; KT, kidney transplant; cGFR, glomerular filtration rate; MDRD, modification of diet in renal disease; CsA, cyclosporine A; NA, not available*.

### Patients under BLT Treatment Present High Frequency of Circulating CD4^+^CD45RA^+^ T Lymphocytes

**Figure 1:**
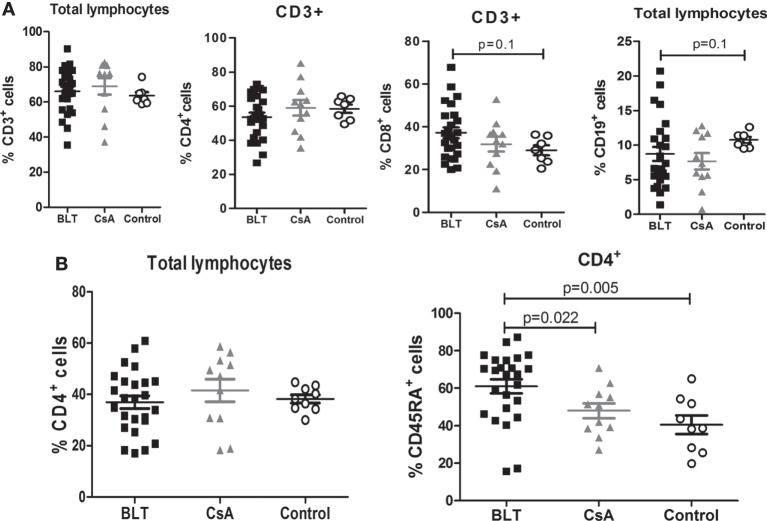
**Increased frequency of naïve CD4^+^ T cells in BLT-treated patients**. **(A)** Normal percentages of CD3^+^, CD4^+^, and CD8^+^ cells are found in BLT- (*n* = 24) and CsA- (*n* = 11) treated patients compared to control (*n* = 9), and a slight reduction of B (CD19^+^) cells is observed in BLT-treated patients. **(B)** Analysis of naïve T cell populations by the expression of CD45RA marker shows a significant increase in the frequency of CD4^+^CD45RA^+^ naïve T cells in BLT compared to CsA-treated kidney transplant patients and controls. Statistical analysis was performed using non-parametric Mann–Whitney test, two tailed. BLT, belatacept; CsA, Cyclosporine A. Control group was composed by healthy individuals.

No significant differences were found between the frequencies of peripheral lymphocytes (CD3^+^, CD3^+^CD4^+^, CD3^+^CD8^+^, and CD19^+^ cells) among patients with BLT and CsA compared with controls (Figure 1A). Although the frequency of CD4^+^ T cells was similar between all groups (Figure 1B, left), patients on long-term BLT treatment presented significantly higher percentages of circulating CD4^+^CD45RA^+^ T lymphocytes compared with CsA and control groups (BLT = 61.04 ± 3.74; CsA = 48.03 ± 3.97; control = 40.56 ± 4.92; *p* < 0.05) (Figure 1B, right).

### Decreased Frequency of FOXP3^+^ and Reduced Expression of FOXP3 and CD25 Inside of Total CD4^+^ T Cells from BLT-Treated Patients

**Figure 2:**
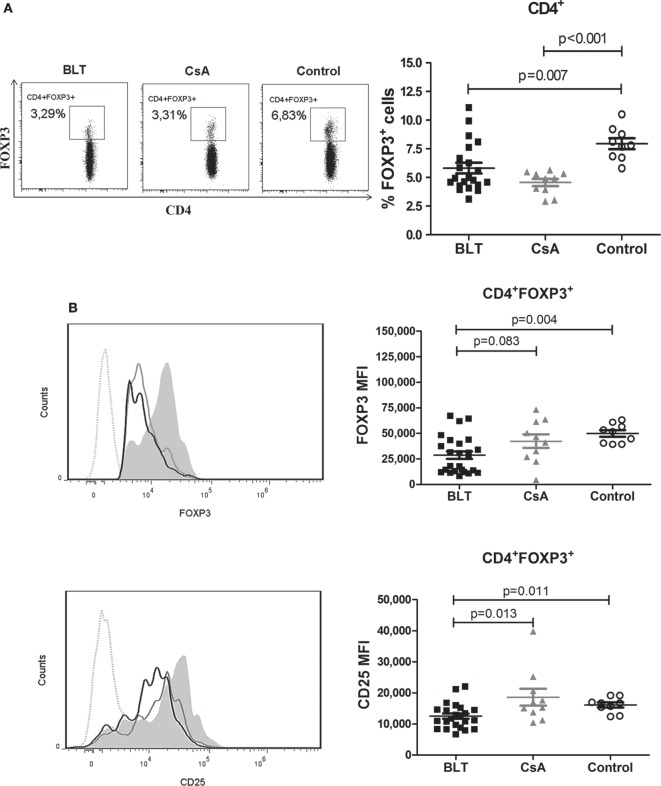
**CD4^+^ T cells from BLT-treated patients show a diminished frequency of FOXP3^+^ subset with reduced levels of FOXP3 and CD25**. **(A)** A reduced frequency of CD4^+^FOXP3^+^ T cells was found in patients under BLT (*n* = 21) and CsA (*n* = 10) treatment compared to controls (*n* = 9). Lefts panels show representative dot plots from patients and controls. **(B)** Levels of FOXP3 and CD25 expression within the CD4^+^ subpopulations, were calculated as mean fluorescence intensity (MFI), showing a significant reduction in BLT-treated patients compared to CsA and control group. Left panels show representative histograms from BLT (black solid line), CsA (gray solid line), and control (filled histogram) individual; dotted line denotes fluorescence minus one control. MFI values from each individual were calculated by subtracting the MFI values of the non-expressing subpopulation for each marker from the corresponding expressing subpopulation. Statistical analysis was performed using non-parametric Mann–Whitney test, two tailed. BLT, belatacept; CsA, Cyclosporine A. Control group was composed by healthy individuals.

To examine the effect of BLT versus CsA on the frequency of circulating Tregs in kidney transplant patients, we first analyzed the expression of FOXP3 in total CD4^+^ T cells. Within the CD4^+^ T cell population, the percentage of FOXP3^+^ cells in both transplanted patients was significantly reduced compared with control group (BLT = 5.81 ± 0.47; CsA = 4.57 ± 0.32; control = 7.94 ± 0.48; *p* < 0.01) (Figure 2A). In addition, the expression [mean fluorescence intensity (MFI)] of FOXP3 in CD4^+^FOXP3^+^ T cells from BLT-treated patients was lower than those of CsA and controls groups (BLT = 28,731 ± 3,639; CsA = 42,362 ± 6,687; control = 49,964 ± 3,108; *p* < 0.005) (Figure 2B, upper panel). As high expression of CD25 characterizes Tregs, we next analyzed CD25 within the CD4^+^ FOXP3^+^ T cells population and found that this marker was significantly reduced in BLT-treated patients compared with CsA and control groups. There was no difference of CD25 MFI between CsA and controls (BLT = 12,509 ± 839.1; CsA = 18,612 ± 2,728; control = 16,111 ± 808.6; *p* < 0.05) (Figure 2B, lower panel).

### Frequency of Peripheral CD4^+^CD25^hi^CD127^lo/−^ Tregs from Kidney Transplant Patients Are Reduced and Express Lower Levels of FOXP3 and CD25

**Figure 3:**
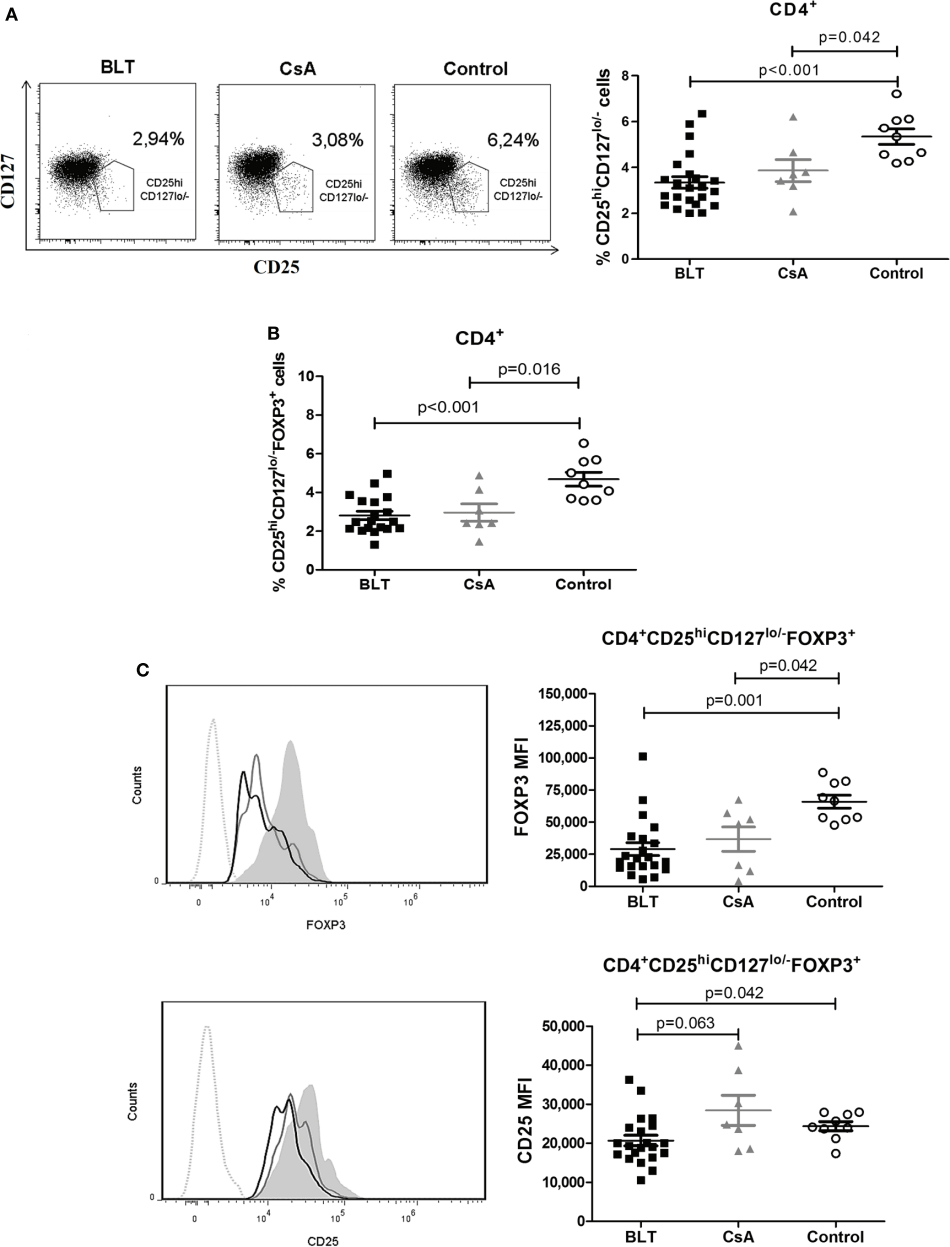
**Peripheral CD4^+^CD25^hi^CD127^lo/−^FOXP3^+^ T cells [regulatory T cells (Tregs)] from kidney transplant patients showed a reduction in frequency and expression levels of FOXP3**. **(A)** Reduced frequency of CD4^+^CD25^hi^CD127^lo/−^ T cells in long-term kidney transplant patients (BLT = 12 and CsA = 7) versus controls (*n* = 9). Right panels show representative dot plots from patients and controls. **(B)** Reduction in the percentage of CD4^+^CD25^+^CD127^lo/−^FOXP3^+^ T cells in compared to controls. **(C)** Expression of markers characteristic of the Treg phenotype demonstrates a significant reduction of FOXP3 and CD25 in Tregs from BLT-treated patients. Left panels show representative histograms from BLT (black solid line), CsA (gray solid line), and control (filled histogram) individual; dotted line is a fluorescence minus one control. Mean fluorescence intensity (MFI) values from each individual were calculated by subtracting the MFI values of the non-expressing subpopulation for each marker from the corresponding expressing subpopulation. Statistical analysis was performed using non-parametric Mann–Whitney test, two tailed. BLT, belatacept; CsA, Cyclosporine A; control group was composed by healthy individuals.

Some reports indicate that a CD25^hi^ and CD127^lo/−^ phenotype on CD4^+^ T cells allows more accurately to distinguish Tregs from activated CD25^+^ conventional T cells, particularly in human samples (16). As shown in Figure 3A, the proportion of CD25^hi^CD127^lo/−^ T cells within the CD4^+^ region was significantly reduced in both BLT- and CsA-treated patients compared with the control group (BLT = 3.31 ± 0.25; CsA = 3.83 ± 0.51; control = 5.28 ± 0.36; *p* < 0.05). As a result, the frequency of CD25^hi^CD127^lo/−^FOXP3^+^ T cells among CD4^+^ cells was significantly reduced in both treatment groups compared with controls (BLT = 2.81 ± 0.22; CsA = 2.97 ± 0.44; control = 4.69 ± 0.36; *p* < 0.05) (Figure 3B). In addition, the expression of FOXP3 in CD4^+^CD25^hi^CD127^lo/−^FOXP3^+^ T cells of transplanted patients was lower than those of controls (BLT = 29,099 ± 5,020; CsA = 36,840 ± 9,520; control = 65,997 ± 5,026; *p* < 0.05) (Figure 3C, upper panel). Expression of CD25 within the CD4^+^CD25^hi^CD127^lo/−^FOXP3^+^ population was significantly reduced in BLT-treated patients compared with CsA and control groups (BLT = 20,701 ± 1,363; CsA = 28,447 ± 3,859; control = 24,389 ± 1,154; *p* < 0.05) (Figure 3C, lower panel).

**Figure 4:**
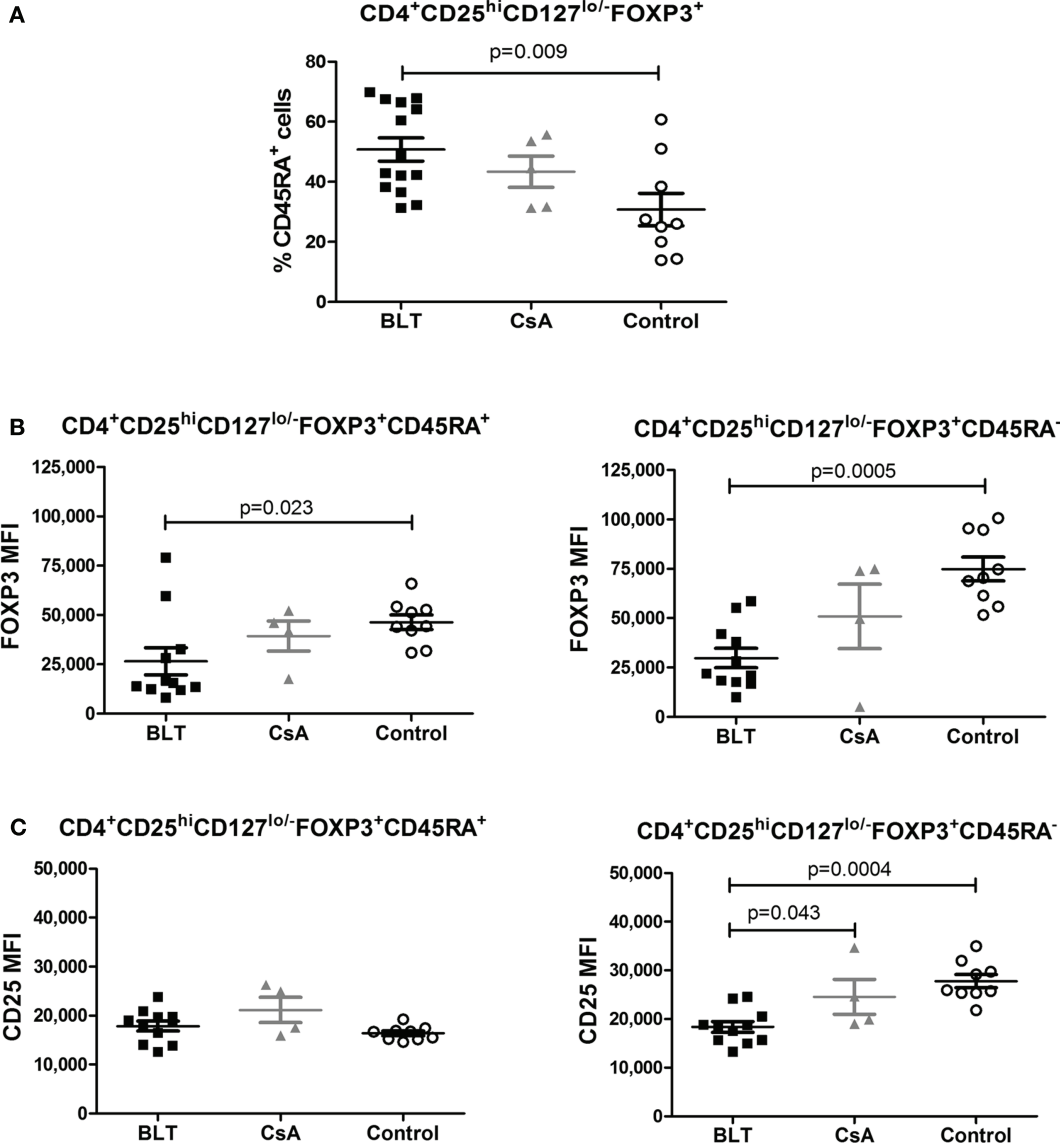
**Reduced FOXP3 expression on BLT regulatory T cells (Tregs) is not a consequence of the accumulation of Tregs with naïve phenotype**. Patients under BLT treatment showed an increase frequency of naïve Tregs **(A)**; however, a reduced expression of FOXP3 **(B)** was found in both naïve (CD45RA^+^) and activated (CD45RA^−^) Tregs from patients under BLT (*n* = 14) treatment compared to CsA (*n* = 4) and controls (*n* = 9). **(C)** CD25 expression was significantly reduced in activated but not naïve Tregs from BLT patients. Statistical analysis was performed using non-parametric Mann–Whitney test, two tailed. BLT, belatacept; CsA, Cyclosporine A. Control group was composed by healthy individuals.

Moreover, in order to examine whether the reduced expression of FOXP3 and CD25 is the result of changes in the composition of the total Treg pool, we determined the frequency of CD45RA^+^ and Helios^+^ within the CD4^+^CD25^hi^CD127^lo/−^FOXP3^+^ T cells of kidney transplant patients and controls. Similarly to what was observed for total CD4^+^ T cells, BLT-treated patients showed a significantly increased frequency of CD4^+^CD25^hi^CD127^lo/−^ FOXP3^+^CD45RA^+^ T cells (“naïve” Tregs) compared with control group (BLT = 50.76 ± 3.89; CsA = 43.34 ± 5.19; control = 30.78 ± 5.41; *p* = 0.009) (Figure 4A). Interestingly, both CD45RA^+^ and CD45RA^−^ Tregs from BLT-treated patients display lower levels of FOXP3 compared to the controls (Figures 4B,C), so that our results are not a consequence of the accumulation of Tregs with naïve phenotype.

**Figure 5:**
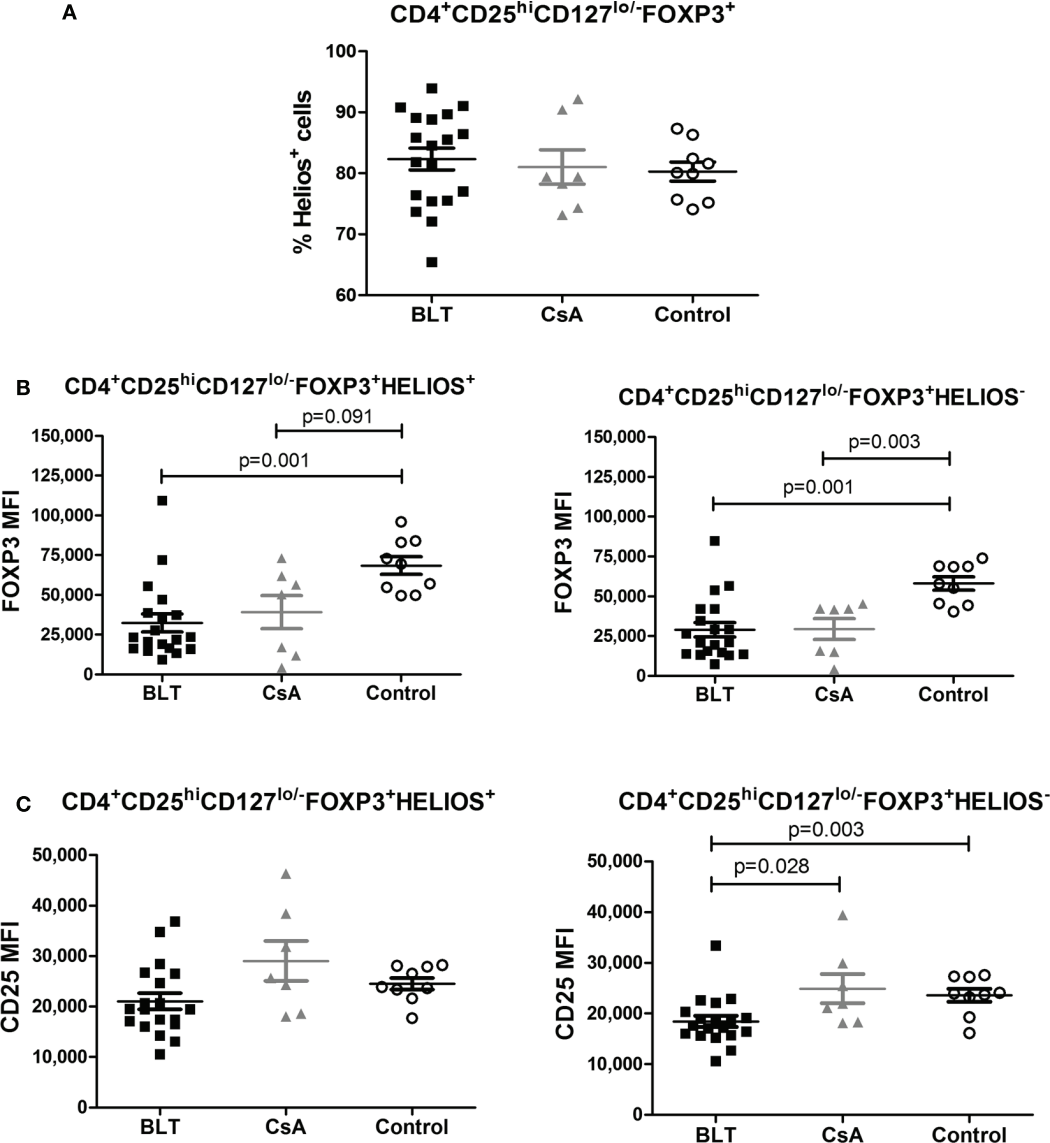
**FOXP3 expression is reduced in regulatory T cells (Tregs) from BLT-treated patients independently of Helios expression**. **(A)** No differences in the frequency of Helios^+^ on Tregs population from the three evaluated groups. **(B)** Reduced expression of FOXP3 within Helios^+^ (left panel) or Helios^−^ Tregs (right panel) of transplanted patients (BLT = 19 and CsA = 7) compared with control group (*n* = 9). **(C)** CD25 expression was significantly reduced in Helios^−^ but not in Helios^+^ Tregs from BLT patients. Statistical analysis was performed using non-parametric Mann–Whitney test, two tailed. BLT, belatacept; CsA, Cyclosporine A. Control group was composed by healthy individuals.

No differences between groups were observed regarding the frequency of Helios^+^ Tregs (Figure 5A) within the CD4^+^CD25^hi^CD127^lo/−^FOXP3^+^ subpopulation. Furthermore, FOXP3 was similarly downregulated in BLT-treated patients among Helios^+^ and Helios^−^ Tregs compared to controls (Figures 5B,C). This transcription factor has been recently shown to cooperate with FOXP3 to promote Treg suppression function, suggesting that it could be a useful marker for the identification of stable Foxp3 (+) Tregs that are primarily generated in the thymus (tTregs) (17). Therefore, FOXP3 and CD25 expression is reduced in Tregs from BLT-treated patients independently of Helios expression.

Interestingly, within the CD4^+^CD25^hi^CD127^lo/−^ region, we found that only patients under CsA treatment showed a reduced percentage of FOXP3^+^ cells compared to controls (76.80 ± 2.83 versus 88.46 ± 1.20; *p* = 0.002); while the percentage of FOXP3^+^ cells within this region was not significantly altered in kidney transplant patients under BLT treatment compared to controls (83.29 ± 2.39 versus 88.46 ± 1.20; *p* = 0.07). Furthermore, some CsA-treated patients (three out of seven) showed a proportion of FOXP3^+^ cells outside the characteristic CD4^+^CD25^hi^CD127^lo/−^ region (Figure S1 in Supplementary Material).

### Tregs from BLT-Treated Patients Display Impaired Suppressive Function

**Figure 6:**
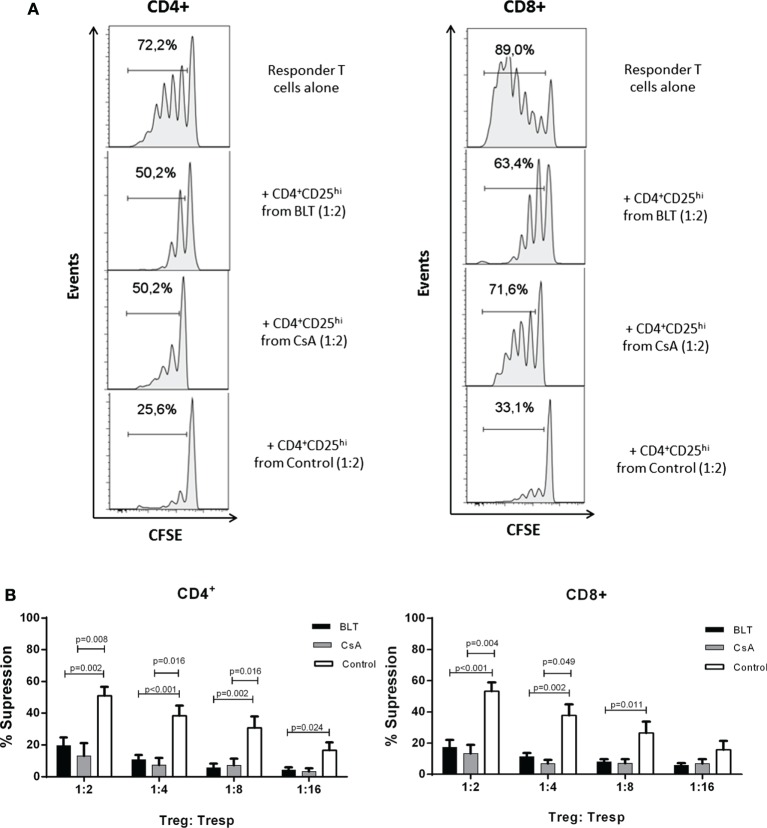
**Impaired suppressive function of regulatory T cells (Tregs) from kidney transplant patients**. **(A)** Histograms show cells proliferation of carboxy fluorescein succinimidyl ester (CFSE)-labeled CD3^+^ T cells in the absence (responder T cells alone) or presence of Tregs (CD4^+^CD25^veryhi^ gated) from kidney transplant patients (BLT and CsA) or healthy subjects (control), numbers indicate the percentage of dividing cells. One representative experiment is presented. **(B)** CD4^+^CD25^hi^ T cells (Treg) isolated from BLT- (black bars, *n* = 9) or CsA- (gray bars, *n* = 4) treated patients showed impaired suppression of both autologous responder CD4^+^ and CD8^+^ T cell (Tresp) proliferation at all evaluated Tregs:Tresp ratios, compared to controls (white bars, *n* = 8). There were no significant differences between Tregs from BLT- and CsA-treated patients. Suppression was calculated as relative inhibition using the following formula: [(Tresp proliferation without Tregs − Tresp proliferation with Tregs)/Tresp proliferation without Tregs] × 100. Statistical analysis was performed using non-parametric Mann–Whitney test, two tailed. BLT, belatacept; CsA, Cyclosporine A.

To determine whether the long-term immunosuppressive therapies had any effect in Treg function, CD4^+^CD25^hi^ T cells were purified from each group for *in vitro* suppression assays. As the percentage of CD25^+^FOXP3^+^ cells within the CD4^+^ subpopulation was reduced in kidney transplant patients (Figure S2 in Supplementary Material), for the suppression assays a gate was set to sort Tregs containing >85% of CD4^+^CD25^hi^FOXP3^+^ T cells similar to the control group (CD4^+^CD25^veryhi^ gate, Figure S3A in Supplementary Material). CD4^+^CD25^hi^ T cells from controls were able to suppress proliferation of both autologous CD4^+^ and CD8^+^ T cells at all evaluated ratios. By contrast, CD4^+^CD25^hi^ T cells isolated from CsA- and BLT-treated patients showed reduced suppression even at high ratios of Tregs to CD4^+^ T cells (BLT = 19.52 ± 5.16; CsA = 17.74 ± 6.81; control = 51.03 ± 5.52 at 1:2 ratio; *p* < 0.01) and CD8^+^ T cells (BLT = 17.31 ± 4.83; CsA = 21.67 ± 3.20; control = 53.31 ± 5.66 at ratio 1:2 ratio; *p* < 0.01) (Figure 6), being associated with the reduced levels of FOXP3 and CD25 observed in the isolated CD4^+^CD25^hi^ T cells of all kidney transplant patients (Figure S3B in Supplementary Material). The suppressive capacity of Tregs was not significantly different between BLT- and CsA-treated patients. Of note, in the absence of Tregs, T cells from patients were equally able to proliferate after anti-TCR stimulation (Figure S4 in Supplementary Material).

### Tregs from BLT-Treated Patients Slightly Reduced IFN-γ by Activated T Cells

**Figure 7:**
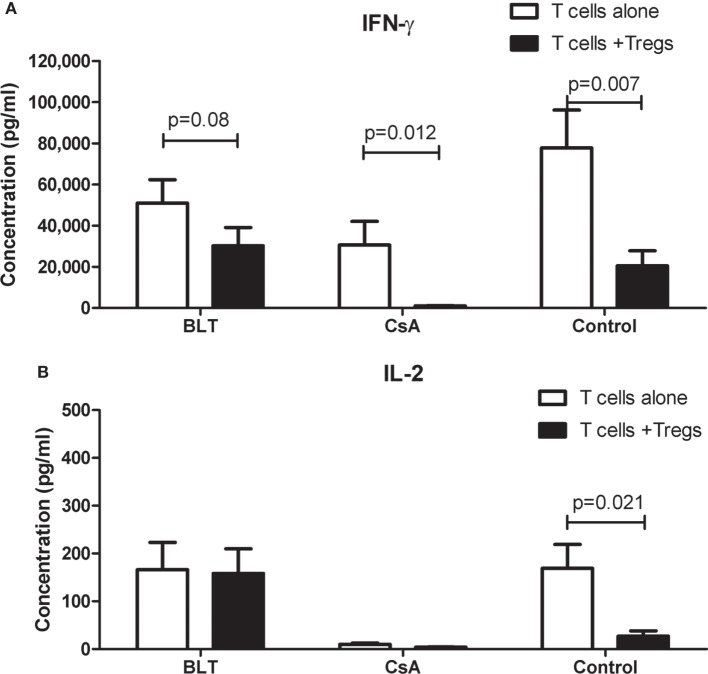
**IFN-γ and IL-2 production in suppression cocultures from kidney transplant patients**. Cytokines were measured in the supernatant cultures of suppression assays from kidney transplant patients (BLT = 3 and CsA = 4) and controls (*n* = 4), after a stimulus with beads CD3/CD28 (responder T cells alone) or cocultured with autologous regulatory T cells (Tregs) by 4 days. **(A)** Responder T cells alone from transplanted individuals produce IFN-γ after CD3/CD28 stimulus in the same way of controls, and this production is reduced in the presence of autologous Tregs. Tregs from BLT-treated patients slightly reduced IFN-γ production by activated T cells. **(B)** Alterations in IL-2 production were observed in transplanted individuals compared with controls. Responder T cells alone from BLT but not CsA-treated patients produce normal levels of IL-2. Statistical analysis was performed using non-parametric Mann–Whitney test, two tailed. BLT, belatacept; CsA, Cyclosporine A. Control group was composed by healthy individuals.

To investigate whether the impaired ability of Tregs from patients to inhibit T cell proliferation was accompanied by a reduced production of cytokines by responders T cells, we analyzed the cytokine secretion in the culture supernatants of the same suppression assays. As shown in Figure 7A, we found a decrease in the production of IFN-γ in the cocultures compared to responders T cells alone when patient’s Tregs were used. This reduction was lower in BLT-treated patients to that observed in cultures of CsA and controls. Interestingly, although the production of IL-2 in responder T cells alone was similar between controls and BLT-treated patients, the levels of IL-2 were not significantly reduced in the cocultures from these patients compared to controls (Figure 7B). By contrast, a reduced production of IL-2 was found in responders T cells alone and cocultures from CsA-treated patients.

### FOXP3 TSDR Is Methylated in Tregs from BLT- and CsA-Treated Patients

**Figure 8:**
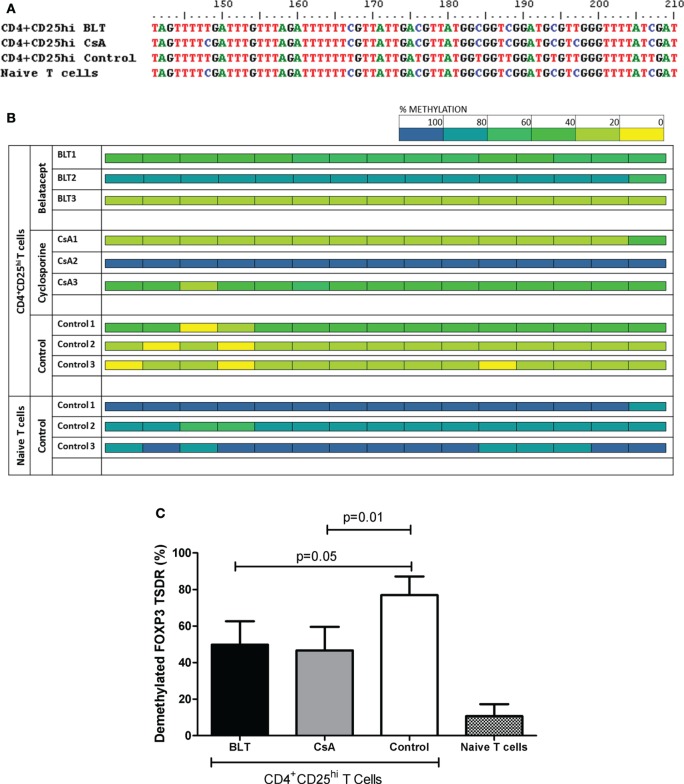
**Altered methylation in the regulatory T cell (Treg) FOXP3 TSDR from kidney transplant patients**. Sequence analysis of the TSDR region of the FOXP3 gene from BLT, CsA, or controls (three male individuals per group, five clones per individual). **(A)** After bisulfite treatment, all demethylated CpG cytosines (blue) convert to thymines (red), as seen with CD4^+^CD25^hi^ Tregs from the control (CD4^+^CD25^veryhi^ gated). By contrast, naïve T cells display a completely methylated TSDR and were used as negative control. Data from representative individuals from each group are shown. **(B)** The panel shows the methylation status of the TSDR FOXP3 in the indicated T cells from the three evaluated groups (three individuals per group). Each square represents one CpG analyzed, data are presented as mean percentage methylation of five clones per individual. Methylation color code ranges from yellow (0% methylation) to blue (100% methylation) according to the color scale (upper right). **(C)** Percentage of demethylation from each evaluated group, represented as the ratio between the numbers of demethylated cytosines and the total number of sequenced CpG sites within the TSDR region.

It has been previously reported that stable expression of FOXP3 is important to maintain Treg function (18), therefore we next analyzed the methylation state of the FOXP3 TSDR from isolated CD4^+^CD25^hi^ T cells of transplanted patients and controls, which were previously used for suppression assays (CD4^+^CD25^veryhi^ gate, Figure S3A in Supplementary Material). As FOXP3 gene is located into X chromosome, we evaluated only male individuals and the results were reported as percentage of methylated CpG (of a total of 15) from five positive clones obtained from each individual. When we analyzed the percentage of methylation from all CpG sites sequenced, we observed a variability in kidney transplant patients, where some patients were almost all methylated (BLT2 and CsA2) and others showed a full demethylation (BLT3 and CsA1) independently of immunosuppressive treatment, compared to controls where almost all clones sequenced were demethylated (Figure 8B). As a result, the complete analysis of all patients showed a significant increase in the percentage of methylation in the FOXP3 TSDR in Tregs from BLT- and CsA-treated patients compared to controls (Figure 8C). This enhanced methylation could be associated with the reduced of suppression observed in Tregs from transplanted patients (Figure 6).

## Discussion

Regulatory T cells have been implicated in tolerance induction to allogeneic organ transplantation (13). Thus, it is important to ensure that immunosuppressive drugs do not interfere in Treg development and/or function (19). Studies have reported that kidney transplant patients under BLT treatment show a better renal function and less chronic nephropathy in comparison to those under CsA treatment (4, 6, 20). Some studies claim that Treg frequency in peripheral blood and transplanted organs are increased in patients under BLT treatment (9, 21); however, other studies claim the opposite (12). Such differences may be caused by inconsistent parameters used for the characterization of the total Treg pool, different gating strategies and small numbers of participants in many studies. In the present study, we found no differences in the frequency of Treg (CD4^+^CD25^hi^CD127^lo/−^FOXP3^+^), between patients under BLT and CsA treatment, although they were lower than those found in healthy individuals. In this study, we used, besides the CD25^+^ and FOXP3^+^ markers, the low expression of CD127 to identify Tregs that were evaluated in 35 patients with a mean time of 7 years since transplantation. By contrast, analysis of Tregs in other studies was based on CD25 and FOXP3 expression (9), two markers that are transiently expressed in activated humans T cells (22), and other results were limited by the small numbers of patients included in the study (21).

Helios was initially reported as a marker of Treg generated in the thymus and was used to differentiate tTregs from those generated in the periphery (23). However, recent reports have shown that Helios can be expressed in peripherally induced Tregs and is associated with T cell activation and cell division (24). Interestingly, it was reported that FOXP3^+^Helios^+^ Tregs represent a much more stable population as they differ from FOXP3^+^Helios^−^ Tregs in their epigenetic status of the FOXP3 locus and their capacity to produce effector cytokines (25). Our results suggest that Helios expression is not responsible for the levels of FOXP3, as this marker was similarly downregulated in Helios^+^ and Helios^−^ Tregs from kidney transplant patients, compared to controls (Figure 5). Similarly, an accumulation of “naïve” Tregs cannot account for the reduced expression of FOXP3 on Tregs from BLT-treated patients, as it was affected in both “naïve” (CD45RA^+^) and “activated” (CD45RA^−^) Tregs (Figure 4).

It has been argued that the immunosuppressive drugs used to maintain graft acceptance in kidney transplant patients may be affecting both the development, homeostasis, and function of Tregs (26); BLT blocks T cell signaling by inhibiting the CD28 costimulatory pathway while CsA inhibits the synthesis of NFAT-dependent cytokines such as IL-2. Previous studies have shown that both CD28 and IL-2 pathways signaling are essential for sustained expression of FOXP3 in Treg cells and maintenance of a stable phenotype in the periphery (27, 28). In this study, transplanted patients showed a lower MFI of FOXP3, which is associated with lower suppressor function *in vitro* (Figure 6).

Besides of blocking proliferation, Tregs are able to inhibit the production of cytokines and prevent the differentiation of immune cells. We observed that Tregs from BLT-treated patients slightly reduced IFN-γ production despite their impaired suppressive function on T cell proliferation (Figure 7A). In this context, it has been reported that Tregs can inhibit IFN-γ production without blocking the proliferation of CD4^+^ T cells (29). Moreover, their suppressor effects on TCR signaling, IL-2/IFN-γ transcription as well as IFN-γ production were retained in conventional T cells after the removal of Tregs (30). So, it is probable that Tregs require a more prolonged cellular contact to be able in suppress the proliferation of immune cells (31). A reduction or alteration of membrane markers as CTLA-4 or defects in expression of receptors to IL-2 (CD25) could explain the lack of suppression of proliferation observed in our transplanted patients. In this context, Tregs from BLT-treated patients showed diminished CD25 expression (Figure 3C) which could affect the “consumption” of IL-2 by Tregs, explaining the lack of IL-2 depletion observed in the cocultures with Tregs from these patients (Figure 7B).

Many reports have claimed the “division of labour” between distinct Treg subpopulations in the maintenance of tolerance (32). In the context of allospecific tolerance, it has been claimed that tTregs may participate in the inhibition of T-effector cell trafficking to the target organ while antigen-specific iTregs primarily prevent T-cell priming by acting on antigen-presenting dendritic cells (33). Therefore, it would be relevant to analyze the impact of inmunosuppressors in the percentage and/or function of both subpopulations. Our data showed a decrease in percentage and suppressor function of circulating Tregs; however, no specific markers are currently available to reliably distinguish thymic versus peripherally induced Treg populations (24).

Although there is a previous study claiming that neither BLT nor CsA affect the normal function of Treg cells (11), the current study analyzed the patient’s Treg suppressor capacity on responder T cells from the same kidney transplant patients, while the former one used responder T cells from their respective donors. Interestingly, we were unable to detect strong direct alloreactive responses from our transplanted patients toward their donors APCs, even in the presence of exogenous IL-2 (data not shown), which suggests a possible deletion of direct allospecific T cells. In addition, in long-term kidney transplant patients as those included in this study, alloreactive T cells may likely recognize alloantigens by an indirect pathway (34), and thus suppression function of Tregs on alloreactive T cells activated by this pathway should be pursued. Therefore, we cannot exclude the possibility that Tregs cells with an indirect pathway of allospecificity may have a normal suppressor function. It is not clear whether the relevant allospecific Treg subpopulation of long-term kidney transplant patients can be found in peripheral blood or rather it has migrated to the transplanted organ to accomplish its function, as has been suggested (8).

Our study showed that some transplanted patients display a low frequency and decreased expression of FOXP3 within CD4^+^CD25^hi^CD127^lo/−^ region (Figure 3), which was previously reported for CsA-treated patients (35). In addition to affecting the generation or homeostasis of the Treg pool, this reduction may be caused by the fact that some Tregs might lose its FOXP3 expression due to the action of the immunosupressors on FOXP3 stability, that is controlled by epigenetic and post-translational modifications (36, 37). In this context, demethylated TSDR (Treg-specific demethylation region) of the FOXP3 gene is indicative of stable FOXP3 expression and Treg lineage commitment (36). Unlike other studies which analyzed the methylation state of total PBMC by real time PCR (38), here we decided to sequence the whole TSDR region of sorted Tregs to obtain a more complete information about the epigenetic status of the FOXP3 TSDR in this subpopulation. Interestingly, our data showed that transplanted patients under BLT or CsA treatment presented partial methylation of the CpG islands present in the TSDR (Figure 8), which is associated with the impaired suppressor function from Tregs of these patients. Thus, the loss of demethylation in TSDR region might be responsible for the reduced FOXP3 expression observed in these patients, and lower levels of FOXP3-dependent markers such as CD25 and CTLA-4 (39, 40), where its upregulation is associated with enhanced Treg activity (28, 41). It has been reported that ten–eleven translocation (TET) enzymes might participate in the demethylation of FOXP3 (42). TET enzymes catalyzes the conversion of 5-methylcytosine (5mC) to 5-hydroxymethylcytosine that are intermediates in the process of DNA demethylation. High expression of these enzymes was observed in thymic FOXP3^+^ Treg subsets and a deletion of TET enzymes led to FOXP3 hypermethylation (43). In addition, high expression of TET enzymes have been observed after TCR signaling and IL-2 was necessary to maintain their expression (44) and, it was shown that (IL-2)-activated Stat5 facilitated Tet1 and Tet2 binding to FOXP3. On other hand, CD28 signaling in Tregs is required for stabilization of FOXP3 mRNA in thymic Treg precursors (45). Therefore, the block of these pathways by immunosuppressive drugs could explain the methylation in the FOXP3 TSDR observed in transplanted patients under BLT and CsA treatment.

The variability found in the percentages of GpG island demethylation from the TSDR region of patients under immunosuppressive treatment suggests that the impaired suppressor function of Tregs from transplanted patients may also be caused by FOXP3-independent factors, such as IL-10, TGFβ, granzyme B, and galectin-1 (46). Therefore, although our results are suggestive of the impact of BLT and CsA on TSDR methylation, it is necessary to include other phenotypic and functional analysis to determine the mechanism underlying the alterations of Tregs in long-term transplanted patients.

Our findings suggest that circulating Tregs are no solely responsible for the quiescence immune state achieved in stable long-term patients. In this context, a recent study showed BLT-treated patients have increased cellular populations with regulatory function such that IL-10^+^ B cells and IDO^+^ cells (9, 47), which could promote tolerance during transplantation and facilitate the long-term survival and function of allografts.

In summary, this study has shown that even in patients with stable graft function, treatment with BLT can have an advertent effect on Treg, suggesting that the beneficial effects reported by BLT cannot be only explained by circulating Tregs. However, we cannot exclude the immunomodulatory role of Tregs, as allospecific Treg function has not directly been evaluated. Interestingly, a report showed that combination of T cell depletion, BLT, and Sirolimus favored Treg expansion and abrogation of T-cell alloreactive anti-donor responses *in vitro*, although the study was performed only at 12 months of transplantation (48). Whether the drug effects on Tregs have an impact on the ultimate outcome of renal allografts remains unclear. Therefore, the effects of long-term immunosuppression on Tregs should be taken into account in the design of new Treg-based therapy.

## Author Contributions

GS supervised the research, analyzed the data, wrote the manuscript, and obtained funding. EA-S performed experiments, analyzed data, and wrote the manuscript. AC-H, GA-M, and JRR-A performed experiments and revised manuscript. JA designed the study, obtained patient consentment to participate, followed-up the patients, and reviewed the manuscript. FR-T contributed with reagents and revised manuscript. VCS contributed with reagents. EC, MG, IB, and EH-M gathered clinical information and blood samples. EM-U, GM-R, MV, and DR obtained patient consentment to participate, followed-up the patients, and reviewed the manuscript. EG-Z provided reagents and analyzed data, and reviewed the manuscript.

## Conflict of Interest Statement

The authors declare that the research was conducted in the absence of any commercial or financial relationships that could be construed as a potential conflict of interest. The reviewer FP and handling editor declared their shared affiliation, and the handling editor states that the process nevertheless met the standards of a fair and objective review.

## Abbreviations

CNI, calcineurin inhibitors; CsA, Cyclosporine A; BLT, belatacept; Tregs, regulatory T cells; FOXP3, Forkhead box P3; IL-2, interleukin 2; APC, antigen-presenting cell; CTLA-4, cytotoxic T-lymphocyte-associated antigen 4; PBMCs, Peripheral blood mononuclear cells; CFSE, Carboxy fluorescein succinimidyl ester; FBS, fetal bovine serum.
