# Supplementary figures and images for: Methylation of FOXP3 TSDR Underlies the Impaired Suppressive Function of Tregs from Long-term Belatacept-Treated Kidney Transplant Patients
Source: Front Immunol. 2017 Mar 3;8:219. doi: 10.3389/fimmu.2017.00219 (PMC5334349; doi:10.3389/fimmu.2017.00219)

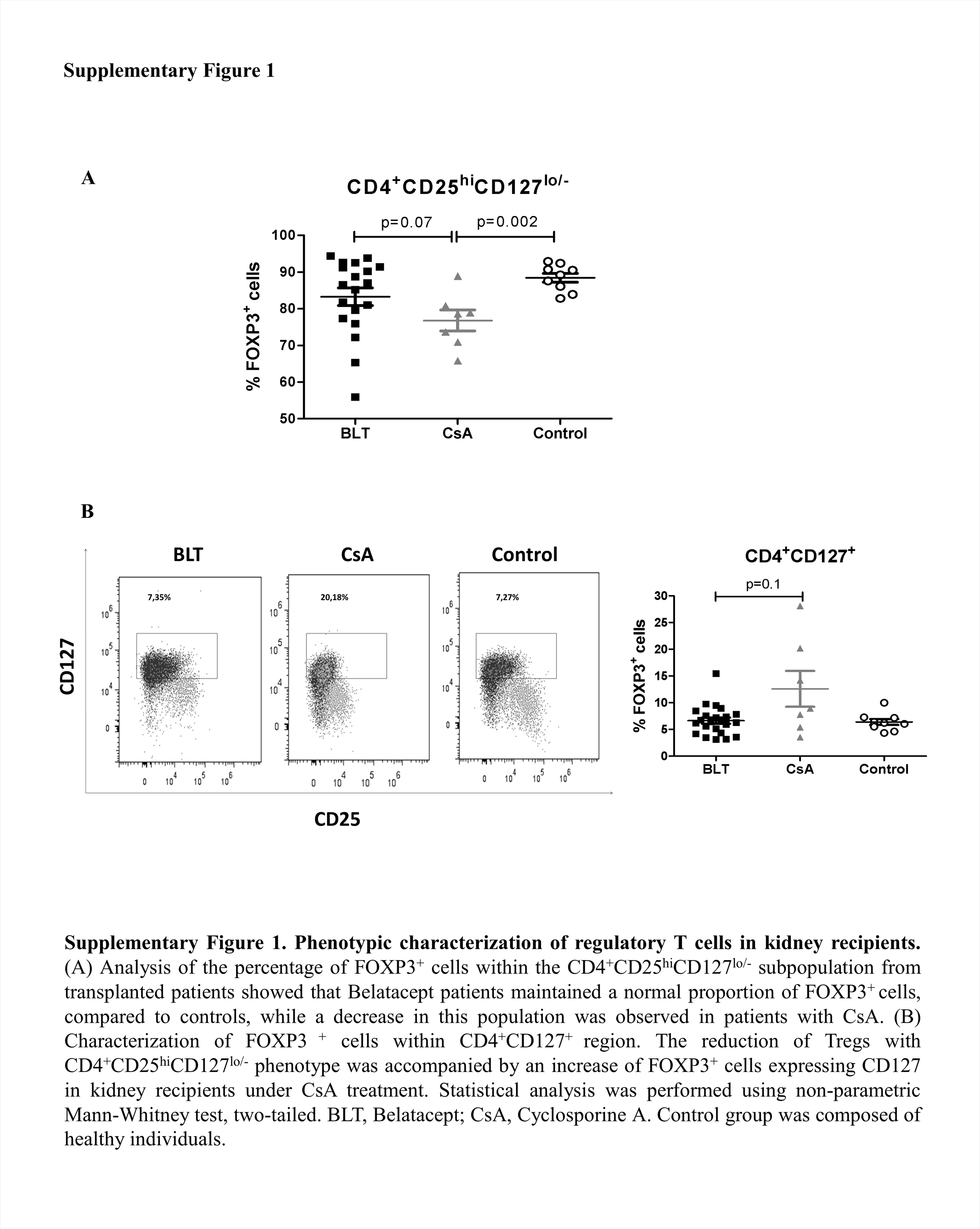

Supplement: Supplementary file 1 [file Image_1.TIF]

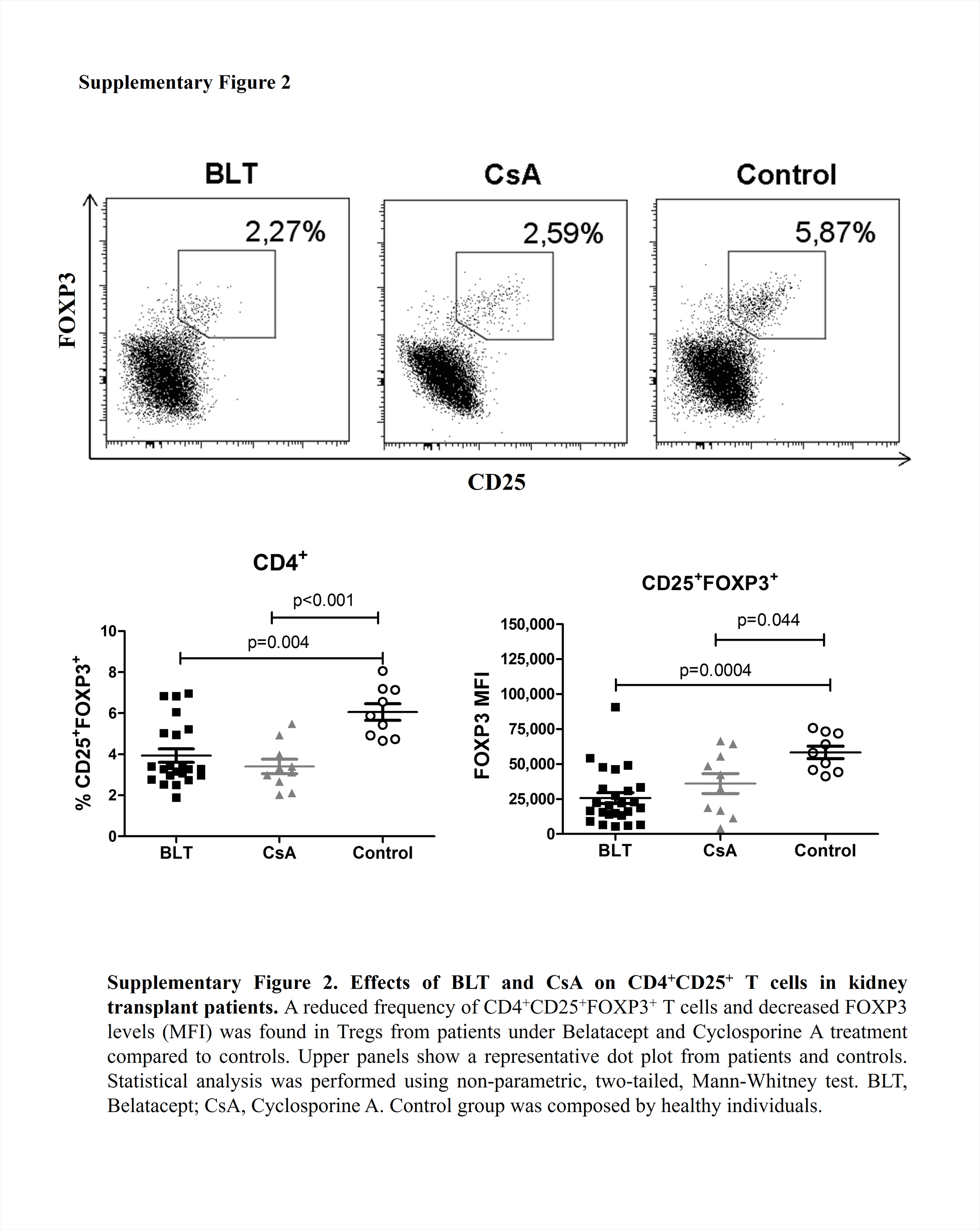

Supplement: Supplementary file 2 [file Image_2.TIF]

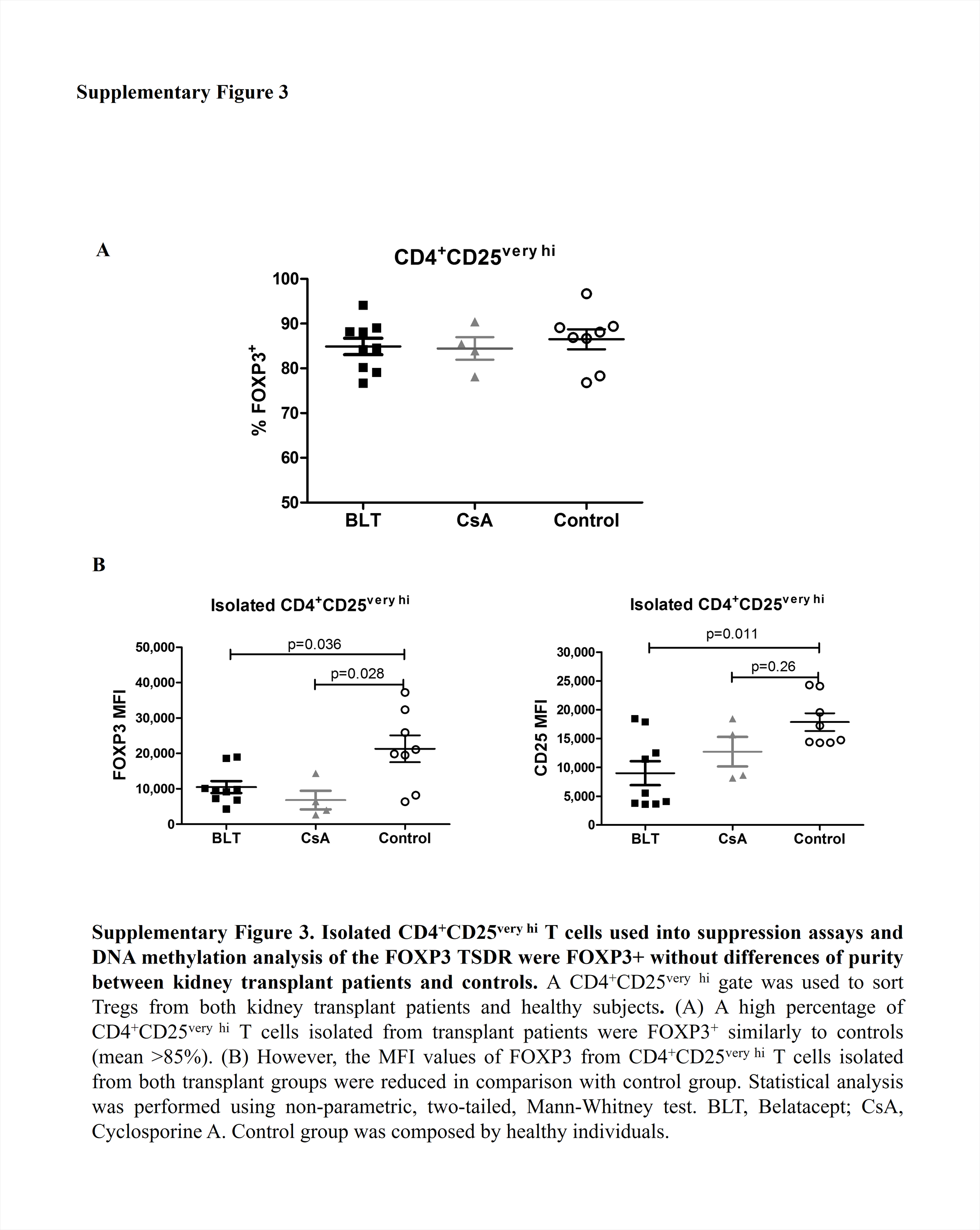

Supplement: Supplementary file 3 [file Image_3.TIF]

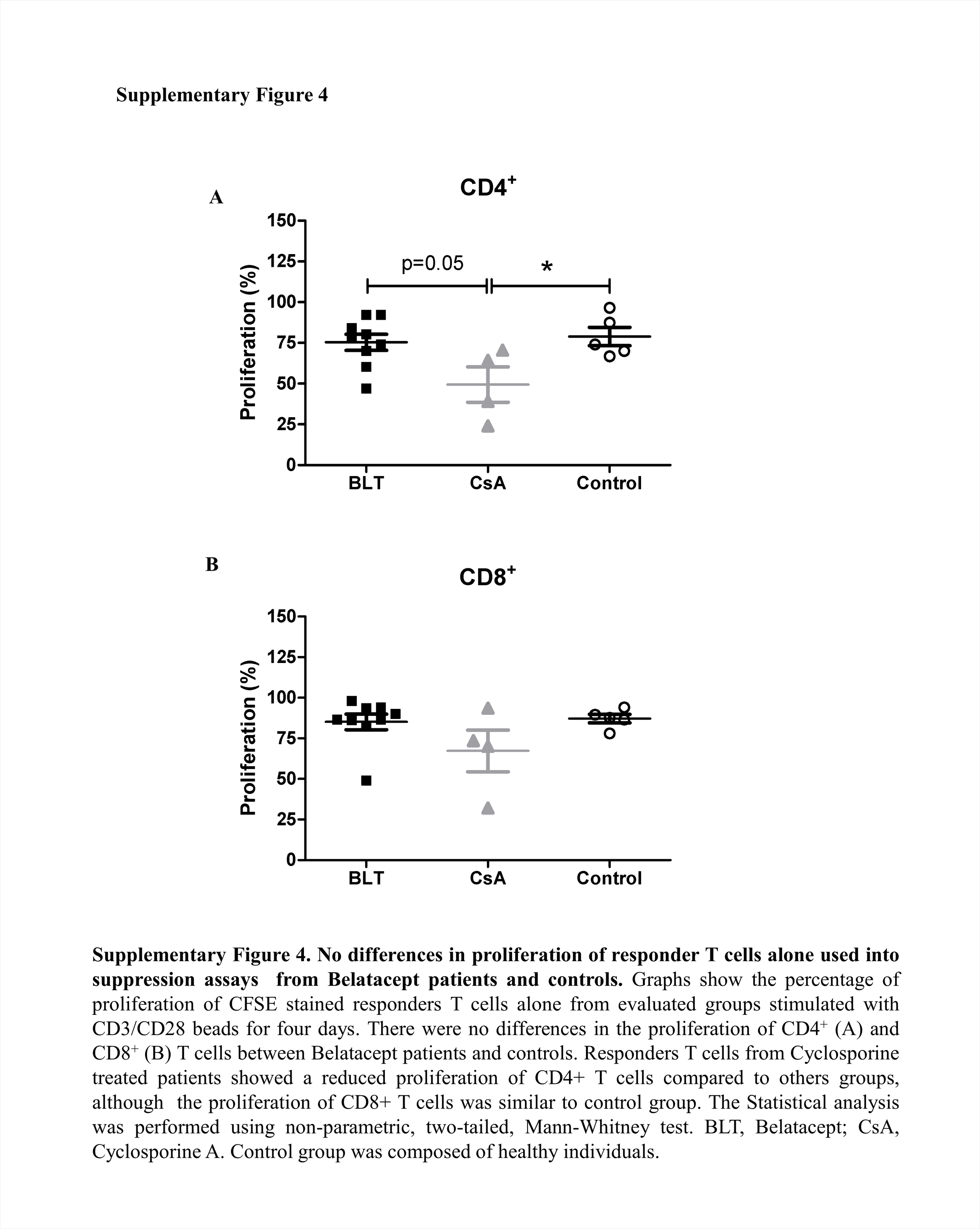

Supplement: Supplementary file 4 [file Image_4.TIF]
